# Polygenic risk scores across the extended psychosis spectrum

**DOI:** 10.1038/s41398-021-01720-0

**Published:** 2021-11-26

**Authors:** Lukasz Smigielski, Sergi Papiol, Anastasia Theodoridou, Karsten Heekeren, Miriam Gerstenberg, Diana Wotruba, Roman Buechler, Per Hoffmann, Stefan Herms, Kristina Adorjan, Heike Anderson-Schmidt, Monika Budde, Ashley L. Comes, Katrin Gade, Maria Heilbronner, Urs Heilbronner, Janos L. Kalman, Farahnaz Klöhn-Saghatolislam, Daniela Reich-Erkelenz, Sabrina K. Schaupp, Eva C. Schulte, Fanny Senner, Ion-George Anghelescu, Volker Arolt, Bernhard T. Baune, Udo Dannlowski, Detlef E. Dietrich, Andreas J. Fallgatter, Christian Figge, Markus Jäger, Georg Juckel, Carsten Konrad, Vanessa Nieratschker, Jens Reimer, Eva Reininghaus, Max Schmauß, Carsten Spitzer, Martin von Hagen, Jens Wiltfang, Jörg Zimmermann, Anna Gryaznova, Laura Flatau-Nagel, Markus Reitt, Milena Meyers, Barbara Emons, Ida Sybille Haußleiter, Fabian U. Lang, Thomas Becker, Moritz E. Wigand, Stephanie H. Witt, Franziska Degenhardt, Andreas J. Forstner, Marcella Rietschel, Markus M. Nöthen, Till F. M. Andlauer, Wulf Rössler, Susanne Walitza, Peter Falkai, Thomas G. Schulze, Edna Grünblatt

**Affiliations:** 1 Department of Child and Adolescent Psychiatry and Psychotherapy, Psychiatric University Hospital Zurich, University of Zurich, Zurich, Switzerland; 2 The Zurich Program for Sustainable Development of Mental Health Services (ZInEP), Psychiatric University Hospital Zurich, Zurich, Switzerland; 3 Institute of Psychiatric Phenomics and Genomics (IPPG), University Hospital, LMU Munich, Munich, Germany; 4 Department of Psychiatry and Psychotherapy, University Hospital, LMU Munich, Munich, Germany; 5 Department of Psychiatry, Psychotherapy and Psychosomatics, Psychiatric University Hospital Zurich, University of Zurich, Zurich, Switzerland; 6 Department of Psychiatry and Psychotherapy I, LVR-Hospital, Cologne, Germany; 7 Department of Neuroradiology, University Hospital Zurich, Zurich, Switzerland; 8 Department of Biomedicine, Human Genomics Research Group, University Hospital and University of Basel, Basel, Switzerland; 9 Institute of Human Genetics, University of Bonn, School of Medicine & University Hospital Bonn, Bonn, Germany; 10 Department of Psychiatry and Psychotherapy, University Medical Center Göttingen, Göttingen, Germany; 11 International Max Planck Research School for Translational Psychiatry, Max Planck Institute of Psychiatry, Munich, Germany; 12 Department of Psychiatry and Psychotherapy, Mental Health Institute, Berlin, Germany; 13 Institute for Translational Psychiatry, University of Münster, Münster, Germany; 14 Department of Psychiatry, University of Münster, Münster, Germany; 15 Department of Psychiatry, Melbourne Medical School, The University of Melbourne, Melbourne, VIC Australia; 16 The Florey Institute of Neuroscience and Mental Health, The University of Melbourne, Parkville, VIC Australia; 17 AMEOS Clinical Center Hildesheim, Hildesheim, Germany; 18 Center for Systems Neuroscience (ZSN), Hannover, Germany; 19 Department of Psychiatry and Psychotherapy, Tübingen Center for Mental Health (TüCMH), University of Tübingen, Tübingen, Germany; 20 Karl-Jaspers Clinic, European Medical School Oldenburg-Groningen, Oldenburg, Germany; 21 Department of Psychiatry II, Ulm University, Bezirkskrankenhaus Günzburg, Günzburg, Germany; 22 Department of Psychiatry, Ruhr University Bochum, LWL University Hospital, Bochum, Germany; 23 Department of Psychiatry and Psychotherapy, Agaplesion Diakonieklinikum, Rotenburg, Germany; 24 Department of Psychiatry, Klinikum Bremen-Ost, Bremen, Germany; 25 Department of Psychiatry, University Medical Center Hamburg-Eppendorf, Hamburg, Germany; 26 Department of Psychiatry and Psychotherapeutic Medicine, Research Unit for Bipolar Affective Disorder, Medical University of Graz, Graz, Austria; 27 Clinic for Psychiatry, Psychotherapy and Psychosomatics, Augsburg University, Medical Faculty, Bezirkskrankenhaus Augsburg, Augsburg, Germany; 28 Department of Psychosomatic Medicine and Psychotherapy, University Medical Center Rostock, Rostock, Germany; 29 Clinic for Psychiatry and Psychotherapy, Clinical Center Werra-Meißner, Eschwege, Germany; 30 German Center for Neurodegenerative Diseases (DZNE), Göttingen, Germany; 31 iBiMED, Medical Sciences Department, University of Aveiro, Aveiro, Portugal; 32 Psychiatrieverbund Oldenburger Land gGmbH, Karl-Jaspers-Klinik, Bad Zwischenahn, Germany; 33 Department of Genetic Epidemiology in Psychiatry, Central Institute of Mental Health, Medical Faculty Mannheim, University of Heidelberg, Mannheim, Germany; 34 Centre for Human Genetics, University of Marburg, Marburg, Germany; 35 Institute of Neuroscience and Medicine (INM-1), Research Center Jülich, Jülich, Germany; 36 Department of Neurology, Klinikum rechts der Isar, School of Medicine, Technical University of Munich, Munich, Germany; 37 Department of Psychiatry and Psychotherapy, Charité Universitätsmedizin, Berlin, Germany; 38 Laboratory of Neuroscience (LIM 27), Institute of Psychiatry, Universidade de São Paulo, São Paulo, Brazil; 39 Neuroscience Center Zurich, University of Zurich and ETH Zurich, Zurich, Switzerland; 40 Zurich Center for Integrative Human Physiology, University of Zurich, Zurich, Switzerland; 41 Department of Psychiatry and Behavioral Sciences, SUNY Upstate Medical University, Syracuse, USA; 42 Department of Psychiatry and Behavioral Sciences, Johns Hopkins University School of Medicine, Baltimore, MD USA

**Keywords:** Clinical genetics, Schizophrenia, Bipolar disorder, Prognostic markers

## Abstract

As early detection of symptoms in the subclinical to clinical psychosis spectrum may improve health outcomes, knowing the probabilistic susceptibility of developing a disorder could guide mitigation measures and clinical intervention. In this context, polygenic risk scores (PRSs) quantifying the additive effects of multiple common genetic variants hold the potential to predict complex diseases and index severity gradients. PRSs for schizophrenia (SZ) and bipolar disorder (BD) were computed using Bayesian regression and continuous shrinkage priors based on the latest SZ and BD genome-wide association studies (Psychiatric Genomics Consortium, third release). Eight well-phenotyped groups (*n* = 1580; 56% males) were assessed: control (*n* = 305), lower (*n* = 117) and higher (*n* = 113) schizotypy (both groups of healthy individuals), at-risk for psychosis (*n* = 120), BD type-I (*n* = 359), BD type-II (*n* = 96), schizoaffective disorder (*n* = 86), and SZ groups (*n* = 384). PRS differences were investigated for binary traits and the quantitative Positive and Negative Syndrome Scale. Both BD-PRS and SZ-PRS significantly differentiated controls from at-risk and clinical groups (Nagelkerke’s pseudo-*R*^2^: 1.3–7.7%), except for BD type-II for SZ-PRS. Out of 28 pairwise comparisons for SZ-PRS and BD-PRS, 9 and 12, respectively, reached the Bonferroni-corrected significance. BD-PRS differed between control and at-risk groups, but not between at-risk and BD type-I groups. There was no difference between controls and schizotypy. SZ-PRSs, but not BD-PRSs, were positively associated with transdiagnostic symptomology. Overall, PRSs support the continuum model across the psychosis spectrum at the genomic level with possible irregularities for schizotypy. The at-risk state demands heightened clinical attention and research addressing symptom course specifiers. Continued efforts are needed to refine the diagnostic and prognostic accuracy of PRSs in mental healthcare.

## Introduction

Psychosis is a mental condition characterized by disturbed contact with reality, affecting cognition, feelings, and behavior. It manifests primarily as sensory experiences in the absence of physical stimuli (hallucinations) and holding bizarre, irrational, or false beliefs (delusions) [1]. While psychotic symptoms are associated with different medical conditions, they typically occur in schizophrenia (SZ), a prototypical psychotic disorder, and as a frequent characteristic in bipolar disorder (BD). Notably, SZ and BD are highly heritable, with heritability estimates of 60–80% [2] and substantially overlapping genetic architectures [3–5]. Psychotic symptoms in SZ and BD also show clinical responses to antipsychotic medication [6]. Despite extensive research, the etiology of psychotic disorders remains unclear, with multiple putative determinants and risk-conferring factors [7]. Within this complex scenario of poly-causation, genetic burdens may predispose certain individuals to developing clinical syndromes in critical life periods [8]. Addressing the heterogeneity of SZ and BD genetics results, a recent investigation assessed the contribution of common variants to disease susceptibility [9]. In addition, the availability of large datasets enabled by high-throughput screening and genomic advances have facilitated the development of polygenic risk scores (PRSs). PRSs cumulatively estimate genome-wide effects of common variants en masse instead of the effects of individual single-nucleotide polymorphisms (SNPs) [10]. In the context of psychosis research, the value of PRSs has been substantiated across numerous applications, including their capacity to differentiate between cases and controls [11, 12] and to predict longitudinal illness courses, diagnostic subtype shifts [13], and responses to antipsychotic medication [14, 15].

Overt psychosis in SZ is often preceded by early signs and mild symptoms developing 2–5 years before a formal diagnosis [16]. Newer diagnostic manuals, such as Diagnostic and Statistical Manual of Mental Disorders, Fifth Edition [17] and International Classification of Diseases, Eleventh Revision [18] incorporate dimensional aspects. A growing body of evidence also supports a continuum-like or extended psychosis spectrum model (cf. categorical approaches) of increasing symptom severity and persistence [19, 20]. These findings are corroborated by the modulating effect of PRS for SZ (SZ-PRS) on well-established neurocognitive intermediate phenotypes, such as prefrontal dysregulation in connection to working memory load, which has been observed in both SZ patients and healthy persons [21]. The polygenic burden indexed by SZ-PRS was also shown to be associated with cognitive–emotional, behavioral, and social impairments, known antecedents of SZ, across the developmental trajectory in large population-based cohorts [22]. Additionally, separate and joint PRSs for SZ and BD (BD-PRS) have proven useful in dissecting BD subtypes [23] and predicting progression to BD and psychotic disorders from a diagnosis of unipolar disorder [24]. As early recognition of psychosis may mitigate its negative individual and societal impacts, recent research has emphasized the importance of early detection and intervention [25]. The concepts of both schizotypy (trait-like psychosis proneness) and at-risk mental state (imminent, basic symptom criteria) have substantially informed preventive research [26, 27]. Nevertheless, the role of schizotypy in the developmental psychopathology of psychosis remains debated [26, 28], and most individuals at high risk do not transition to a diagnostic entity even years after an initial clinical presentation [29]. The molecular and genetic foundation of a broad psychosis spectrum is unclear, yet its exploration may reveal biological common denominators. In this context, the ultimate objectives of PRS analyses include the accurate prediction of disease onset and determination of tailored treatment strategies [10]. However, studies aimed at identifying associations between PRS and quantitative psychotic symptom manifestations have yielded both significant [30, 31] and inconsistent findings [32]. Notably, one study identified a linear relationship only when both cases and controls were pooled [33], underscoring the value of the continuum model. Inconsistencies may also have arisen from possible temporal symptom fluctuations and/or differences in operationalization among the clinical instruments employed. Furthermore, recent genome-wide association studies (GWASs) based on increasing discovery sample sizes and refined algorithms have contributed to the improved PRS power [34], which itself is sufficient motivation for new predictive studies.

This work investigates the associations and between-group differences of PRS computed for SZ and BD across the extended psychosis spectrum. In an early study utilizing PRS [35], SZ-PRS and BD-PRS were significantly associated with SZ and BD diagnostic spectrums. When split into separate diagnoses, SZ-PRS was associated with SZ, schizoaffective disorder (SZA), psychosis not otherwise specified, and BD type I, while BD-PRS was associated with BD type I and BD type II. However, no significant differences between any portion of the spectrum or specific groups were found after controlling for multiple comparisons (Tukey method). In contrast, the present investigation expands the phenotypic poles of normatively diagnosed disorder groups (SZ, BD type I, BD type II, SZA) and controls into the subclinical categories of high risk for developing psychosis, as well as schizotypy. If individuals across these groups differentially share genetic liability, a smooth gradient and severity-related differences in PRS should be observed. Moreover, in a transdiagnostic manner, this work examines the relationship between PRS and a well-validated continuous symptom measure, the Positive and Negative Syndrome Scale (PANSS) [36], using quantile regression modeling [37].

## Methods and materials

### Phenotype definition

Data included in this work come from three main projects, two completed in Switzerland [38, 39], the Zurich Program for Sustainable Development of Mental Health Services (ZinEP) project on at-risk mental state for psychosis and the Exceptional Experiences (EE) project on psychotic-like experiences, as well as the multi-center PsyCourse Study [40] on the affective-psychotic spectrum conducted in Germany and Austria. Two other smaller Swiss projects, the Zurich Family-Trio Study and the Zurich OCD Study, served as additional sources of healthy controls [41, 42]. Data from eight groups were analyzed in this study according to the pre-established criteria: SZ, SZA, BD II, BD I, at-risk state for psychosis (RISK), higher schizotypy (SCHIZ H), lower schizotypy (SCHIZ L), and non-psychiatric controls (CTRL). The at-risk categories included basic symptoms, by some conventions known as “high risk” (HR) and “ultra-high risk” (UHR) for psychosis [29]. The specific inclusion criteria were as follows: at least one cognitive–perceptive basic symptom and/or at least two cognitive disturbances based on the Schizophrenia Proneness Interview [43, 44] for HR; at least one attenuated psychotic symptom, and/or at least one brief limited intermittent psychotic symptom based on the Structured Interview for Prodromal Syndromes [45] for UHR. Additionally, the hypomanic symptoms in this group were measured using the Hypomania Checklist (HCL-32) [46]. The median-split of the Schizotypal Personality Questionnaire (SPQ) [47] score was used, as in earlier studies on higher and lower schizotypy [48, 49], with similar cut-offs and mean values. Psychiatric axis I diagnosis in these two groups was excluded using the Mini-International Neuropsychiatric Interview [50]. The diagnoses and assessments were made by clinicians and trained raters. Patients were recruited based on referrals from clinics or patient register queries. Proficiency in German was a criterion for participation. Written informed consent was obtained from all participants and, for participants under 18 years of age, their parents or legal guardians. All the participating projects were approved by the ethics committees of each respective institution (Zurich: KEK-Nr. E63/2009, KEK-Nr. 2011-0423, KEK-Nr. 2010-0340/3, KEK-Nr. 2010-0340; see detailed information on the PsyCourse Study [40] [www.psycourse.de] provided in the Supplementary Methods and Materials) and conducted in compliance with the Declaration of Helsinki. Table S1 (Supplementary Information) lists the primary inclusion criteria for the eight investigated groups. All data were anonymized for analysis.

### Genotyping and genotype imputation

Genomic DNA was isolated from venous whole blood or saliva (for a small subsample of controls) and genotyped using Illumina Infinium Psych-Array BeadChips (Illumina, San Diego, CA, USA). SNP-level quality control (QC) included the removal of variants with a call rate <98%, significant deviations from Hardy–Weinberg equilibrium (HWE) (*p* < 0.001), or minor allele frequency (MAF) < 0.1%. In the individual-level QC, data were removed to eliminate duplicates, sex mismatches, cryptic relatives (PI-HAT > 0.125), samples with an individual genotyping rate >98%, or heterozygosity rate exceeding 3 SD from the mean. Population stratification was assessed using a principal component analysis on the pairwise genomic relationship matrix. Individuals with genotypes not clustered with 1000 Genomes Project EUR super-populations were removed from the analyses (*n* = 47). After QC, pre-phasing was performed using SHAPEIT [51] and imputation was conducted using IMPUTE2 [52]. The phase 3 integrated variant dataset from the 1000 Genomes Project [53] was used as the reference panel. After imputation, variants with a low information score (INFO < 0.9) or frequency (MAF < 1%) were removed. All data were processed with the same analysis pipeline. Figure S1 (Supplementary Information) visualizes the European origin of participants along the first two ancestry PCs with labeled superpopulations.

### PRS computation

The PRS calculations for each individual were based on the risk alleles and the corresponding odds ratios (ORs) from the most recent GWAS of the Psychiatric Genomics Consortium (PGC3) on SZ [54] and BD [55]. The SZ GWAS included 69,369 cases and 236,642 controls, while the BD GWAS included 41,917 cases and 371,549 controls. PRSs were computed using a recently developed Bayesian regression and continuous shrinkage priors method (PRS-CS) [34, 56]. The PRS-CS method exhibited superior prediction accuracy across multiple complex diseases and traits, compared to several other methods, particularly with large training datasets [56]. In this approach, the posterior effect sizes of SNPs are inferred using information from the GWAS summary statistics and an external linkage disequilibrium reference (1000 Genomes Project phase 3, EUR super-populations), through principles of joint multivariate modeling. The global shrinkage parameter phi (*φ*) [57] was automatically learned from the data (PRS-CS-auto; *φ*^1/2^ ~ *C*^+^(0,1), where *C*^+^(0,1) denotes the half-Cauchy distribution). In our study, the automatically estimated global shrinkage parameter was *φ* = 1.8E−04 for SZ and *φ* = 1.3E−04 for BD. PRS-CS allowed us to use a single polygenic score for each discovery trait (SZ and BD, respectively) that considers the polygenicity of the disorder and appropriately models the genetic architecture of SZ and BD. The PRSs were standardized using *z*-score transformation to facilitate interpretation.

### Statistical analysis: PRSs across groups

An analysis of variance (ANOVA) was conducted to assess between-group differences in SZ-PRS and BD-PRS (with group as predictor and PRS as outcome variable), using subject age, sex, and first five genetic ancestry principal components (PCs) to account for population stratification. Pairwise comparisons were corrected per analysis using a Bonferroni correction for 28 tests performed (i.e., the number of pair combinations for each PRS). Additionally, to assess the associations with group/diagnosis and variance explained by PRS, block-wise binary logistic regressions with healthy controls were conducted. For each PRS, two statistical models with case/control status as outcome were compared, one testing the covariates alone (i.e., age, sex, and the five ancestry-specific PCs as a baseline model) and the other testing the covariates with the added PRS (full model). We report the *R*^2^ value as the difference in Nagelkerke’s pseudo-*R*^2^ [58] between these two nested models as an indicator of explained variance. As these comparisons were against the control condition, no further correction was applied in these non-independent tests.

### Statistical analysis: association between PRSs and symptoms

To examine the effects of PRS predicting the symptom PANSS scores (Positive Symptoms, Negative Symptoms, General Psychopathology, and Total Score) across low, medium, and high symptom score distributions, quantile regressions using three tau (τ) values (0.25, 0.50, 0.75) and accounting for age, sex, and the five ancestry-specific PCs were conducted. The quantiles indicate that 25%, 50%, and 75% of the scores, respectively, fall below each corresponding point in the distribution of scores. Quantile regression [37] was chosen over a transformation because of the floor effect of symptom data. This statistical approach estimates the effects at different locations in the criterion distribution with or without normality assumptions being met, where relying on the mean as a measure of centrality under an ordinary least squares model might be less informative or inappropriate [59].

Statistical analyses were performed using the R statistical computing environment (version 4.0.5). Nominal significance was assessed at an α < 0.05 threshold.

## Results

### Study sample

After QC, 1580 samples (883 males; ages 12–78 years) from the initial pool of 1752 genotyped samples, with complete information for study group, sex, and age, were available for analysis, including 6,600,214 genetic markers. PANSS data were accessible for 1134 individuals (622 males). The characteristics and symptom scores of participants are summarized in Table 1. Within the at-risk group, the following numbers of individuals met the partial (overlapping) criteria: 120 (HR or UHR), 111 (HR), 70 (UHR), 50 (HR without UHR), 86 (risk for BD).

**Table 1:** Sample characteristics.

Phenotypic spectrum	Sample size	% male	Mean age (SD)	Mean PANSS score (SD)			
				Positive symptoms	Negative symptoms	General psychopathology	Total score
Schizophrenia (SZ)	384	64.58	40.54 (12.21)	14.60 (5.87)^a^	16.30 (6.82)^a^	30.61 (10.36)^a^	61.51 (20.58)^a^
Schizoaffective disorder (SZA)	86	38.37	45.63 (10.77)	12.53 (5.74)^b^	14.28 (5.76)^b^	28.80 (8.83)^b^	55.60 (17.11)^b^
Bipolar II disorder (BD II)	96	46.88	46.22 (13.80)	8.69 (2.36)^c^	9.82 (3.77)^c^	23.56 (6.82)^c^	42.07 (11.00)^c^
Bipolar I disorder (BD I)	359	54.32	45.16 (12.72)	9.48 (3.41)^d^	10.21 (3.94)^d^	23.23 (6.48)^d^	42.92 (10.94)^d^
At-risk state for psychosis (RISK)	120	60.00	21.23 (5.93)	12.66 (4.13)^e^	14.19 (5.72)^e^	31.47 (8.18)^e^	58.32 (15.87)^e^
Higher schizotypy (SCHIZ H)	113	62.83	31.68 (10.20)	—	—	—	—
Lower schizotypy (SCHIZ L)	117	72.65	32.51 (11.71)	—	—	—	—
Non-psychiatric controls (CTRL)	305	43.93	37.29 (15.22)	7.08 (0.32)^f^	7.13 (0.50)^f^	16.37 (0.83)^f^	30.58 (1.01)^f^
Total sample	1580	55.89	38.89 (14.20)	11.28 (5.16)^g^	12.42 (6.11)^g^	25.96 (9.40)^g^	49.67 (18.67)^g^

Note: The Positive and Negative Syndrome Scale (PANSS) scores were available for the following sample subsets: ^a^*n* = 362, ^b^*n* = 83, ^c^*n* = 89, ^d^*n* = 328, ^e^*n* = 119, ^f^*n* = 153, ^g^*n* = 1134. The assessments were collected during interviews at clinical presentations.

### Between-group differences

**Fig. 1:**
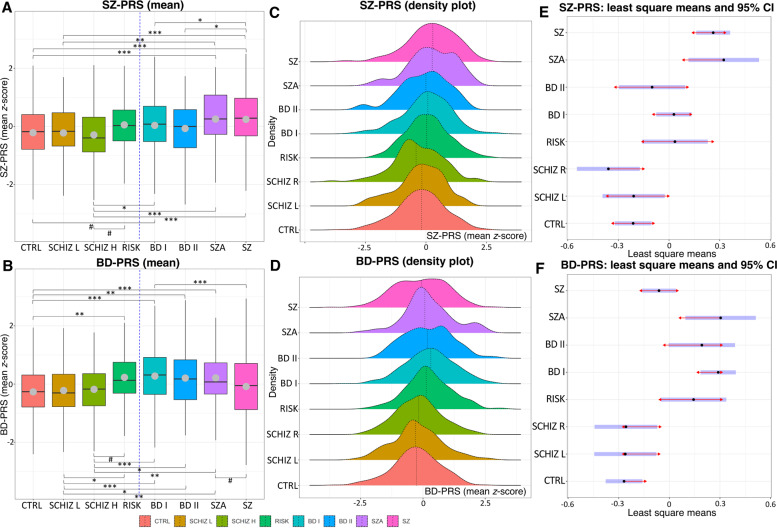
Mean polygenic risk score (PRS) for schizophrenia (SZ-PRS) and bipolar disorder (BD-PRS) in the eight investigated groups. **A**, **B** Each boxplot shows the median (vertical lines) and mean (gray dots) PRS values; whiskers represent the minimum and maximum values; the vertical blue dashed lines separate the groups with subclinical versus clinical symptomatology; brackets with symbols indicate significantly different pairwise Bonferroni-corrected comparisons at *p* < 0.05 (*), *p* < 0.01 (**), and *p* < 0.001 (***) thresholds as well as comparisons trending toward significance (#, adjusted *p* value range, 0.050–0.064). The results are adjusted for age, sex, and the first five ancestry principal components. Higher scores indicate a higher polygenic burden. **C**, **D** The vertical dotted lines within the colored distributions represent the mean PRS for each group. **E**, **F** The blue bars represent confidence intervals for each group’s least squares mean. If the red arrows for two groups overlap, the difference is not significant after a Bonferroni correction. Group abbreviations: BD I bipolar I disorder, BD II bipolar II disorder, CTRL control, RISK at-risk state for psychosis, SCHIZ H higher schizotypy, SCHIZ L lower schizotypy, SZA schizoaffective disorder, SZ schizophrenia.

ANOVA revealed significant among-group differences for both SZ-PRS (*F*_7,1565_ = 9.30, *p* = 2.52 × 10^−11^) and BD-PRS (*F*_7,1565_ = 11.71, *p* = 1.37 × 10^−14^). Out of 28 pairwise Bonferroni-corrected tests for SZ-PRS and for BD-PRS, 9 and 12 were significant, respectively. Figure 1 depicts the mean standardized PRS, density plots, and 95% confidence intervals (CIs) with comparison arrows between groups. Notably, the controls differed from the SZ and SZA groups in SZ-PRS and from the BD I, BD II, SZA, and at-risk groups in BD-PRS. Very similar results were observed for the lower schizotypy group. Additionally, the high schizotypy group differed from most clinical groups and the at-risk group, while the latter was not distinguishable from any clinical diagnoses. Tables 2 and S2 (Supplementary Information) provide detailed statistics for this analysis.

**Table 2:** Main results of the analysis of variance using polygenic risk score (PRS) for schizophrenia (SZ-PRS) and bipolar disorder (BD-PRS) across the eight studied groups (Table S2 provides the full list of pairwise tests).

Measure	ANOVA					Means (z-score)			Significant post hoc tests			
	Sum of squares	df	Mean square	*F*	*p*	Group	Mean	SD	Pairwise contrast	Beta	SE	Adjusted *p*
SZ-PRS	61.01	7	8.716	9.300	2.52 × 10^−11^	CTRL	−0.214	0.988	CTRL–SZA	−0.537	0.121	2.53 × 10^−4^
						SCHIZ L	−0.222	0.947	CTRL–SZ	−0.474	0.076	1.65 × 10^−8^
						SCHIZ H	−0.323	1.018	SCHIZ L–SZA	−0.534	0.146	0.007
						RISK	0.055	0.88	SCHIZ L–SZ	−0.471	0.109	4.50 × 10^−4^
						BD I	0.036	0.986	SCHIZ H–BD I	−0.388	0.112	0.015
						BD II	−0.07	0.977	SCHIZ H–SZA	−0.683	0.146	9.18 × 10^−5^
						SZA	0.253	0.958	SCHIZ H–SZ	−0.621	0.110	5.73 × 10^−7^
						SZ	0.243	1.012	BD I–SZ	−0.233	0.072	0.034
									BD II–SZ	−0.362	0.112	0.034
									CTRL–BD I	−0.242	0.078	0.054^#^
									SCHIZ H–RISK	−0.394	0.129	0.064^#^
Residuals	1466.67	1565	0.937									
BD-PRS	76.51	7	10.930	11.709	1.37 × 10^−14^	CTRL	−0.268	0.943	CTRL–RISK	−0.412	0.113	0.007
						SCHIZ L	−0.222	0.993	CTRL–BD I	−0.558	0.078	2.86 × 10^−11^
						SCHIZ H	−0.181	0.905	CTRL–BD II	−0.461	0.115	0.002
						RISK	0.230	0.905	CTRL–SZA	−0.572	0.12	5.99 × 10^−5^
						BD I	0.276	0.984	SCHIZ L–RISK	−0.404	0.128	0.045
						BD II	0.206	0.96	SCHIZ L–BD I	−0.550	0.111	2.11 × 10^−5^
						SZA	0.213	0.971	SCHIZ L–BD II	−0.453	0.140	0.033
						SZ	−0.095	1.041	SCHIZ L–SZA	−0.565	0.145	0.003
									SCHIZ H–BD I	−0.547	0.112	3.10 × 10^−5^
									SCHIZ H–BD II	−0.450	0.140	0.038
									SCHIZ H–SZA	−0.561	0.146	0.004
									BD I–SZ	0.351	0.072	3.12 × 10^−5^
									SZA–SZ	0.365	0.117	0.050^#^
									SCHIZ H–RISK	−0.401	0.129	0.053^#^
Residuals	1460.92	1565	0.933									

Note: The analyses accounted for age, sex, and the first five genetic ancestry principal components, and statistical significance is indicated by Bonferroni-adjusted *p* values.

*Adjusted p* Bonferroni adjusted *p* value, *beta* beta coefficient, *df* degrees of freedom, *SE* standard error, *BD I* bipolar I disorder, *BD II* bipolar II disorder, *CTRL* controls, *RISK* at-risk state for psychosis, *SCHIZ L* lower schizotypy, *SCHIZ H* higher schizotypy, *SZA* schizoaffective disorder, *SZ* schizophrenia.

^#^Trend toward statistical significance.

### Case–control status

**Fig. 2:**
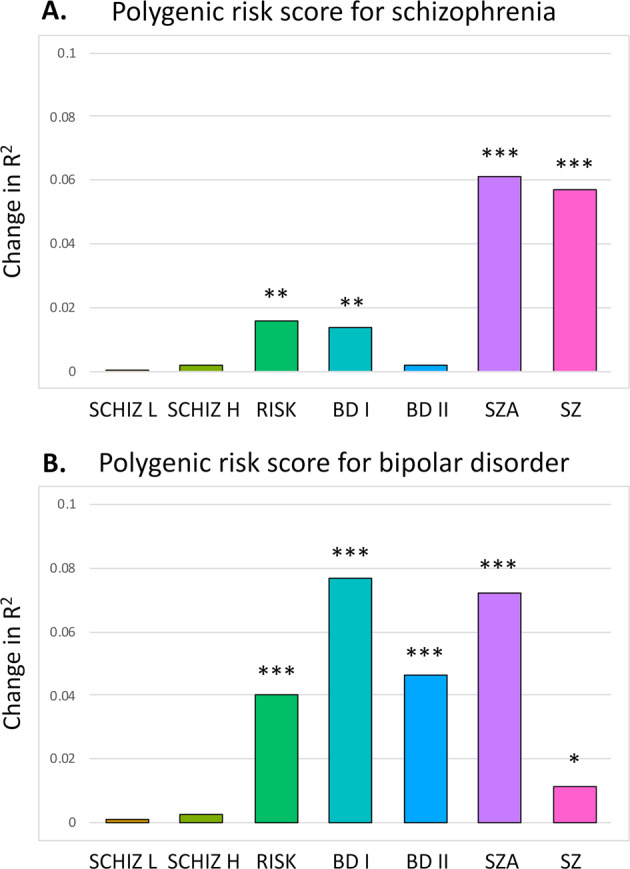
Block-wise binary logistic regressions using polygenic risk score (PRS) in the case–control comparisons. **A** Polygenic risk score for schizophrenia. **B** Polygenic risk score for bipolar disorder. Asterisks indicate statistically significant effects of the full (covariates only) versus baseline (covariates with PRS) models: *p < 0.05; **p < 0.01; ***p < 0.001. Group abbreviations: BD I bipolar I disorder, BD II bipolar II disorder, CTRL controls, RISK at-risk state for psychosis, SCHIZ H higher schizotypy, SCHIZ L lower schizotypy, SZA schizoaffective disorder, SZ schizophrenia.

SZ-PRS was significantly associated with the case–control status in the binary regression for the SZ (odds ratio (OR) = 1.62, 95% CI = 1.37–1.93, *p* = 6.88 × 10^−9^), SZA (OR = 1.82, 95% CI = 1.38–2.45, *p* = 2.04 × 10^−5^), BD I (OR = 1.27, 95% CI = 1.07–1.51, *p* = 0.006), and RISK (OR = 1.57, 95% CI = 1.14–2.20, *p* = 0.006) groups with the controls (change in Nagelkerke’s pseudo-*R*^2^, 1.3–6.1%). BD-PRS was also significantly associated with the case–control status for contrasts involving the BD I (OR = 1.80, 95% CI = 1.51–2.16, *p* = 2.53 × 10^−11^), BD II (OR = 1.65, 95% CI = 1.26–2.18, *p* = 1.98 × 10^−4^), SZA (OR = 1.96, 95% CI = 1.46–2.67, *p* = 3.41 × 10^−6^), SZ (OR = 1.26, 95% CI = 1.10–1.46, *p* = 0.011), and RISK (OR = 2.19, 95% CI = 1.53–3.20, *p* = 8.21 × 10^−6^) groups and the control group, resulting in a slightly higher Nagelkerke’s pseudo-*R*^2^ change range, 4.0–7.7% (Fig. 2 and Table S3 [Supplementary Information]).

### PRSs and symptoms

**Fig. 3:**
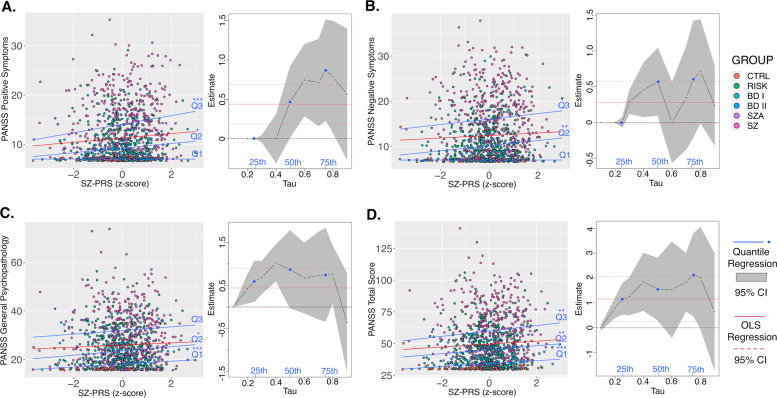
Quantile regression analysis of schizophrenia polygenic risk score (SZ-PRS) predicting symptoms. Three quantiles of symptom score distributions (Q1 = 0.25, Q2 = 0.50, Q3 = 0.75) included three dimensions (**A**–**C**) and the total score (**D**) of the Positive and Negative Syndrome Scale (PANSS). The quantile regression estimates are visualized at 0.1 increments as black lines and with corresponding confidence intervals (CIs) shown as the gray shaded areas. The Q1, Q2, and Q3 quantiles are depicted in blue. For comparison, the ordinary least squares (OLS) regression estimates are shown as red solid lines with their CIs as dashed red lines. Many quantile estimates fell within the CI of the OLS regression slope, indicating overlap (though OLS does not adequately address the floor effect). *p < 0.05; **p < 0.01; ***p < 0.001. There were no significant results for BD-PRS. Group abbreviations: BD I bipolar I disorder, BD II bipolar II disorder, CTRL controls, RISK at-risk state for psychosis, SCHIZ L lower schizotypy, SCHIZ H higher schizotypy, SZA schizoaffective disorder, SZ schizophrenia.

SZ-PRS was found to be a significant predictor of PANSS Total Score at the 25th (*t* = 3.86, *p* = 1.18 × 10^−4^), 50th (*t* = 3.19, *p* = 0.001), and 75th (*t* = 2.64, *p* = 0.008) percentiles. Significant effects were also found for Positive Symptoms at the 50th (*t* = 4.86, *p* = 1.35 × 10^−6^) and 75th (*t* = 2.50, *p* = 0.013) percentiles and for General Psychopathology at the 25th (*t* = 3.41, *p* = 6.70 × 10^−4^) and 50^th^ (*t* = 2.27, *p* = 0.023) percentiles, while Negative Symptoms were significant only at the 50th percentile (*t* = 2.64, *p* = 0.008). In contrast, no significant effects were observed for BD-PRS across any of the PANSS measures. Figure 3 and Table S4 (Supplementary Information) report further details for this analysis.

## Discussion

This work investigated the polygenic burden in four healthy/subclinical groups and four clinical groups. It aimed to quantify the associations and between-group differences of SZ-PRS and BD-PRS and their possible relationship with symptomatic presentation. Both PRSs were differentially useful in elucidating the genome-wide architecture across the extended psychosis spectrum.

Overall, the gradient of genetic risk proximity observed across groups is commensurate with the continuum model [35]. SZ-PRS and BD-PRS performed most robustly for the extremes of the psychosis spectrum, i.e., in distinguishing SZ and BD cases from controls, respectively, corroborating previous findings [11, 12]. Besides, given the significant effects for both PRSs, the existence of shared risk loci for SZA may be inferred from our results relative to this phenotype. Indeed, SZA seems genetically intermediate to BD I and SZ, with evidence from twin studies identifying the same genetic risk factors across SZA, SZ, and manic episodes [60]. Furthermore, similar results were observed for both controls and the low schizotypy group, while the at-risk group seems best positioned close to the clinical spectrum. Moreover, significant associations were seen for both BD type-I (characterized by manic episodes often alternating with depressive episodes) and BD type-II (characterized by hypomania and depression) using BD-PRS, but only for BD type-I using SZ-PRS. While we analyzed roughly four times more BD I than BD II cases, this effect is probably best explained by the putative partial genetic independence within these two BD subtypes [61]. Our findings align with prior reports confirming the ability of PRSs to accurately differentiate subtypes along diagnostic spectra, such as between SZ and other psychotic disorders [11], BD with and without psychotic features [62], and BD with manic and depressive psychosis [63].

Notably, BD-PRS values were higher, conveying a higher polygenic burden, in the at-risk group compared to controls, but not different from those of clinical groups. BD-PRS also explained a significant portion of variance in the binary logistic regression for at-risk status, an effect observed at a somewhat weaker level for SZ-PRS. These findings highlight the inherent genetic vulnerability of this subclinical group. To the best of our knowledge, there has been only one published report of a PRS analysis of a sizeable population of individuals at high risk for psychosis. Perkins et al. [64] recently demonstrated that SZ-PRSs were significantly elevated in individuals transitioning to a psychotic disorder diagnosis within a 2-year interval, compared to unaffected controls, but not in those not transitioning to a clinical diagnosis. Interestingly, our results were significant even without differentiating individuals according to their conversion status. Another key difference is the detection of effects using BD-PRS in our work. In particular, beyond the well-established high clinical risk criteria [29], we also assessed subtle subclinical hypomanic symptoms. A large majority of at-risk individuals (72%) met a cut-off value of ≥14 in the HCL-32 as a secondary measure and tentative risk criterion for BD [38]. This aligns with the common difficulties in unambiguously distinguishing SZ from BD, especially in early disorder onset [65], often continuing as diagnostic instability [66]. Given the large residual variance beyond the currently captured polygenic burden, gene × environment interactions are biologically plausible mechanisms as course specifiers for symptom development [67]. Such an interaction between BD-PRS and childhood trauma predisposing individuals to more severe disorders has indeed been shown recently [68]. As genetic risk is not modifiable, future research should thus also concentrate on psychosocial prevention of mental illness [69].

Our results inform the discussion of psychosis risk enrichment in at-risk individuals [70]. A meta-analysis has estimated a 36% transition rate within 3 years from clinical presentation in such individuals [71]. In the sample included in this study, which was a prospective investigation [38], over the same time window, the transition rate into F20 (schizophrenia), F23 (brief psychotic disorder), and BD diagnoses reached 15%. Currently, risk identification and predictors of exacerbation are primarily based on symptoms, thus lacking proper scalability [72]. In contrast to family history as a proxy for genetic risk, PRS offers a scalable quantitative metric for potential use in clinical contexts conditional on empirical validation [73]. Notably, the severity of psychopathology or clinical deterioration in the psychosis spectrum is often linked to neurocognitive deficits [74] and both SZ-PRS and BD-PRS have repeatedly been linked to cognition [75, 76]. In a differential analysis, most SZ risk alleles were associated with poorer cognition, while most BD risk alleles were linked to better cognition [77]. Accordingly, moderate neurocognitive impairments typically observed in the at-risk state [78], compared to severe impairments in SZ, align with our finding that genetic liability in this group was best characterized by risk variants covered by BD-GWAS. Downstream in the pathway towards symptom development, neurobiological mechanisms influenced by genetic variation [79] might be even more directly associated with emerging cognitive-perceptual disturbances [80]. These brain-based mechanisms may involve altered salience processing, neural coding of prediction error, and precision signaling [81]. Indeed, recent studies combining PRSs with brain imaging parameters have revealed new insights into the genetic determination of brain function and structure in psychosis spectrum [82, 83].

This work also reveals a possible irregularity within the continuum model at the genomic level, suggesting that schizotypy may share less genetic architecture with severe psychotic disorders than formerly thought. This tentative interpretation can be inferred from the lack of significant differences between the high and low schizotypy groups and between either schizotypy group or controls. Notably, in absolute terms, the high schizotypy group had the most negative mean SZ-PRS among all groups and marginally lower SZ-PRS and BD-PRS compared to the at-risk group (trend level). In the binary logistic regressions, neither high nor low schizotypy was significantly associated with either of the two PRSs. It should be emphasized that this sample in our study was composed of healthy participants by excluding individuals with clinical diagnoses. Drawing participants without restriction from a general population could lead to different results. Previously, findings indicating high overlaps between schizotypy and SZ have been made for single candidate genes [84, 85]. Our observation aligns with a recent study by Nenadić et al. reporting no association between SZ-PRS, BD-PRS, or MDD-PRS and symptom SPQ scores in non-clinical subjects [28]. Similarly, another PRS study identified a counter-intuitive negative relationship between SZ-PRS and schizotypal dimensions [86]. These studies challenge the key role of genetic predisposition and emphasize environmental stress as a moderator of schizotypy-related phenotypic expression [86], further informing the discussion on competition versus synergism of genetic–environmental influences [87]. Thus, schizotypy may be better positioned as a qualitatively distinct taxon-like cluster rather than along a unidimensional liability scale [88]. Furthermore, the possibility of “benign” schizotypy [89] supported by some brain-based endophenotype findings [90, 91] suggests that some protective mechanisms may impede a psychotic decomposition of schizotypal traits. While this claim merits further investigation, our results encourage a local reappraisal of the genetic continuum model for schizotypy.

Our findings also contribute to the ongoing discussion of the symptom- and function-level correlates of PRS in support of the continuum model. To the best of our knowledge, this study is the largest to investigate relations between PRS and the gold standard PANSS and is also unique in its transdiagnostic approach. We observed significant associations with SZ-PRS across all symptom measures. However, we were unable to detect any effect whatsoever using BD-PRS, suggesting its lack of specificity in this application and/or its limited power. Previous studies varied in their capacity to find associations with continuous symptom measures. These earlier results included no significant effects [32], links with negative symptoms [92], general psychopathology [30], and a mixture of positive, negative, and cognitive trait dimensions [93]. It has been suggested that a higher SZ-PRS typically predisposes individuals to a general vulnerability [94]. Our results from 1134 data points extend this conclusion based on the observed effect for overall symptomology (PANSS total score) across all three quantiles examined. Still, the strongest effect was identified at the high quantile for specific and more severe positive symptoms, a hallmark of psychosis. Overall, the distribution-dependent effects, especially as linked to different symptom facets and their interactions with environmental factors, may be a source of interesting findings in the field [68, 95].

This study has two particularly promising clinical applications of PRSs in the context of psychotic disorders and their antecedents: as potential support or a stratification tool in early diagnostic decision-making; in informing pre-emptive or full treatment strategies. Substantial clinical and research-based evidence demonstrates the utility of early interventions in reducing the highly debilitating character of psychotic disorders [96]. Indeed, long periods of untreated illness are among the most negative predictors of outcome, progression into chronic forms, and total time spent disabled [97]. Variation in help-seeking behavior may also delay presentation to healthcare specialists [98]. This further motivates the search for objective methods of early detection, which may be as easy as genotyping a blood sample and performing an analysis with a sufficiently large training dataset. For example, integrating PRS with other risk-conveying variables has been shown to improve the prediction of psychosis [64]. Proposals for refining this approach have been incorporated into current pilot projects aiming to create poly-risk scores that consider idiosyncratic combinations of risk and protective factors [99]. PRS-based approaches might also guide drug targeting [100] or explain resistance to pharmacotherapy [15]. For example, PRSs have been shown to be associated with responses to antipsychotic medication [14] and lithium [101]. Such PRS-informed bio-typing may identify patients who will benefit more from one pharmacological treatment among alternatives.

We acknowledge some limitations of this work. The numbers of participants varied substantially among groups. Inclusion of more polarized schizotypy groups (i.e., more extreme phenotypes) might also have been better suited to the goals of this study. The cross-sectional nature of these findings implies the possibility of a progression across the phenotypic spectrum, development of comorbidities, or dampening of genetic liability later in life. Indeed, the at-risk group had the lowest mean age. We also did not consider the role of medication. Finally, as GWASs exclude rare variants, we only investigated the effects of common variants with population frequencies of 1% or more. There are also more general limitations to polygenic risk profiling discussed in the current scientific debate on PRS, which has included articles covering its potential clinical utility [10] and bioethics [102], with some expressly critical tones [103]. Notably, provided the ancestral differences in linkage disequilibrium and polymorphisms, the predictive accuracy of PRS is currently constrained to individuals of European descent, owing to the lack of statistically powerful GWASs of psychiatric disorders and traits from ethnically diverse populations [104].

While PRS is certainly not immediately ready for routine clinical application, this study highlights the value of PRS for SZ and BD in elucidating the genetic architecture of psychosis and identifying vulnerable individuals who may benefit from early attention and monitoring. Future research should focus on possible differential developmental trajectories and factors precipitating or mitigating pre-existing genetic liability.

## Supplementary information

Supplementary Information

## Data Availability

PsyCourse data are available according to a mutually agreed memorandum of understanding. Other data and analysis code are available upon reasonable request.
