# Supplementary material for: Polygenic risk scores across the extended psychosis spectrum
Source: Transl Psychiatry. 2021 Nov 26;11:600. doi: 10.1038/s41398-021-01720-0 (PMC8626446; doi:10.1038/s41398-021-01720-0)
Supplement: Supplementary file 1 — Supplementary Information [file 41398_2021_1720_MOESM1_ESM.pdf]

Supplementary Information for:

Polygenic risk scores across the extended psychosis spectrum

Content:

Supplementary Methods and Materials

Supplementary Tables S1, S2, S3, S4

Supplementary Fig. S1

Supplementary References

Supplementary Methods and Materials: Ethical approval for the "PsyCourse" project

Initially, the project was approved by the Ethics Committee of the University Medical Center Göttingen. Some participating clinical centers were teaching hospitals of the University Medical Center Göttingen and were thus covered by this initial approval. For the remaining clinical sites, an additional approval from the respective Ethics Committees was obtained. These were as follows (clinical centers, project identification codes and dates of approval are reported in brackets): Ethics Committees of the University Medical Center Göttingen (UMG Göttingen, Bad Zwischenahn, Eschwege, Asklepios Specialized Hospital Göttingen, Hildesheim, Lüneburg, Liebenburg, Osnabrück, Rotenburg, Tiefenbrunn, Wilhemshaven; 23/9/10; 3rd of December 2010), Medical Faculty of the LMU Munich (Munich and Augsburg; 17-13; 25th of February 2013), Medical Faculty of the RU Bochum (Bochum; 4644-13; 18th of June 2013), Medical Association Bremen (Bremen Ost; 337; 20th of April 2012), Medical University of Graz (Graz; 25-335 ex 12/13; 13th of June 2013), Ulm University (Günzburg; 236/12; 10th of September 2012) and Medical Association Westfalen-Lippe and Medical Faculty University of Münster (Münster; 2015-011-b-S; 20th of January 2015), Medical Faculty of the University of Tübingen (Tübingen; 096/2013BO1; 19th of June 2013).

Table S1. Primary inclusion criteria for the studied groups.

Group		Phenotypic definition / inclusion criteria	
1.	Schizophrenia (SZ)[a]	DSM-IV diagnosis: 295.10/295.20/295.30/295.60/295.90	Higher end of the spectrum
2.	Schizoaffective disorder (SZA)[a]	DSM-IV diagnosis: 295.70	
3.	Bipolar II disorder (BD II)[a]	DSM-IV diagnosis: 296.89	
4.	Bipolar I disorder (BD I)[a]	DSM-IV diagnosis: 296.x	
5.	At-risk state for psychosis (RISK)[b]	[i] at least one cognitive-perceptive basic symptom (COPER) and/or<br>[ii] at least two cognitive disturbances (COGDIS) and/or<br>[iii] at least one attenuated psychotic symptom (APS) and/or<br>[iiii] at least one brief limited intermittent psychotic symptom (BLIPS).<br><br>• [i–ii] = high-risk (HR) criteria (1), based on the Schizophrenia Proneness Interview (2);<br>• [iii–iiii] = ultra-high risk (UHR) criteria (1), based on the Structured Interview for Prodromal Syndromes (3).	Lower end of the spectrum
6.	Higher schizotypy (SCHIZ H)[c]	• threshold of >19 from the group median-split on the Schizotypal Personality Questionnaire (SPQ total score) (4);<br>• clinical diagnosis was excluded using the Mini-International Neuropsychiatric Interview (5).	
7.	Lower schizotypy (SCHIZ L)[c]	• threshold of ≤19 from the group median-split on the SPQ total score;<br>• clinical diagnosis was excluded using the Mini-International Neuropsychiatric Interview (5).	
8.	Non-psychiatric controls (CTRL)[a,d,e]	• according to the current and past mental health status from the Short Diagnostic Interview for Mental Disorders (6), Structured Clinical Interview for the DSM (SCID) (7) or Schedule for Affective Disorders and Schizophrenia for School-Age Children-Present and Lifetime (K-SADS-PL) (8).	

Notes: [a]PsyCourse Study; [b]ZInEP study; [c]EE study; [d]Zurich Family-Trio study; [e]Zurich OCD study.

Table S2. Results of the pairwise comparisons across eight investigated groups on polygenic risk scores (PRS) for schizophrenia and bipolar disorder.

**Polygenic risk score for schizophrenia (SZ-PRS)**								
**Pairwise contrast**	**Beta**	**SE**	***df***	***T* ratio**	**Lower CI**	**Upper CI**	***Adjusted p***	***p***
CTRL–SCHIZ L	-0.003	0.109	1565	-0.028	-0.344	0.338	1.000	0.978
CTRL–SCHIZ H	0.147	0.110	1565	1.336	-0.197	0.490	1.000	0.182
CTRL–RISK	-0.248	0.113	1565	-2.195	-0.601	0.105	0.793	0.028
CTRL–BDI	-0.242	0.078	1565	-3.106	-0.485	0.002	0.054	0.002
CTRL–BDII	-0.112	0.115	1565	-0.976	-0.473	0.248	1.000	0.329
CTRL–SZA	-0.537	0.121	1565	-4.454	-0.914	-0.160	2.53×10^{-4}	9.03×10^{-6}
CTRL–SZ	-0.474	0.076	1565	-6.232	-0.712	-0.236	1.65×10^{-8}	5.91×10^{-10}
SCHIZ L–SCHIZ H	0.150	0.128	1565	1.168	-0.251	0.551	1.000	0.243
SCHIZ L–RISK	-0.245	0.128	1565	-1.909	-0.646	0.156	1.000	0.057
SCHIZ L–BDI	-0.238	0.111	1565	-2.149	-0.586	0.109	0.891	0.032
SCHIZ L–BDII	-0.109	0.140	1565	-0.781	-0.547	0.329	1.000	0.435
SCHIZ L–SZA	-0.534	0.146	1565	-3.664	-0.989	-0.078	0.007	2.57×10^{-4}
SCHIZ L–SZ	-0.471	0.109	1565	-4.327	-0.812	-0.130	4.50×10^{-4}	1.61×10^{-5}
SCHIZ H–RISK	-0.394	0.129	1565	-3.057	-0.798	0.009	0.064	0.002
SCHIZ H–BDI	-0.388	0.112	1565	-3.466	-0.739	-0.038	0.015	5.42×10^{-4}
SCHIZ H–BDII	-0.259	0.141	1565	-1.843	-0.699	0.181	1.000	0.065
SCHIZ H–SZA	-0.683	0.146	1565	-4.669	-1.141	-0.225	9.18×10^{-5}	3.28×10^{-6}
SCHIZ H–SZ	-0.621	0.110	1565	-5.637	-0.966	-0.276	5.73×10^{-7}	2.05×10^{-8}
RISK–BDI	0.006	0.119	1565	0.051	-0.365	0.377	1.000	0.959
RISK–BDII	0.135	0.146	1565	0.927	-0.321	0.592	1.000	0.354
RISK–SZA	-0.289	0.152	1565	-1.908	-0.763	0.185	1.000	0.057
RISK–SZ	-0.227	0.115	1565	-1.966	-0.587	0.134	1.000	0.049
BDI–BDII	0.129	0.112	1565	1.158	-0.22	0.478	1.000	0.247
BDI–SZA	-0.295	0.117	1565	-2.518	-0.662	0.072	0.333	0.012
BDI–SZ	-0.233	0.072	1565	-3.237	-0.458	-0.008	0.035	0.001
BDII–SZA	-0.424	0.145	1565	-2.93	-0.877	0.029	0.096	0.003
BDII–SZ	-0.362	0.112	1565	-3.239	-0.711	-0.012	0.034	0.001
SZA–SZ	0.062	0.117	1565	0.533	-0.304	0.429	1.000	0.594
**Polygenic risk score for bipolar disorder (BD-PRS)**								
**Pairwise contrast**	**Est**	**SE**	***df***	***T* ratio**	**Lower CI**	**Upper CI**	***Adjusted P***	***P***
CTRL–SCHIZ L	-0.008	0.109	1565	-0.07	-0.348	0.333	1.000	0.944
CTRL–SCHIZ H	-0.011	0.110	1565	-0.102	-0.354	0.332	1.000	0.919
CTRL–RISK	-0.412	0.113	1565	-3.657	-0.764	-0.059	0.007	2.64×10^{-4}
CTRL–BDI	-0.558	0.078	1565	-7.187	-0.801	-0.315	2.86×10^{-11}	1.02×10^{-12}
CTRL–BDII	-0.461	0.115	1565	-4.011	-0.821	-0.101	0.002	6.34×10^{-5}
CTRL–SZA	-0.572	0.120	1565	-4.758	-0.949	-0.196	5.99×10^{-5}	2.14×10^{-6}
CTRL–SZ	-0.207	0.076	1565	-2.726	-0.445	0.031	0.182	0.006
SCHIZ L–SCHIZ H	-0.004	0.128	1565	-0.028	-0.404	0.397	1.000	0.978
SCHIZ L–RISK	-0.404	0.128	1565	-3.16	-0.804	-0.004	0.045	0.002
SCHIZ L–BDI	-0.550	0.111	1565	-4.967	-0.897	-0.204	2.11×10^{-5}	7.54×10^{-7}
SCHIZ L–BDII	-0.453	0.140	1565	-3.246	-0.890	-0.016	0.034	0.001
SCHIZ L–SZA	-0.565	0.145	1565	-3.884	-1.019	-0.110	0.003	1.07×10^{-4}
SCHIZ L–SZ	-0.199	0.109	1565	-1.834	-0.540	0.141	1.000	0.067
SCHIZ H–RISK	-0.401	0.129	1565	-3.111	-0.803	0.002	0.053	0.002
SCHIZ H–BDI	-0.547	0.112	1565	-4.891	-0.897	-0.197	3.10×10^{-5}	1.11×10^{-6}
SCHIZ H–BDII	-0.450	0.140	1565	-3.207	-0.889	-0.011	0.038	0.001

SCHIZ H–SZA	-0.561	0.146	1565	-3.841	-1.018	-0.104	0.004	1.28×10^{-4}
SCHIZ H–SZ	-0.196	0.110	1565	-1.781	-0.540	0.148	1.000	0.075
RISK–BDI	-0.146	0.118	1565	-1.236	-0.516	0.224	1.000	0.217
RISK–BDII	-0.049	0.146	1565	-0.338	-0.505	0.406	1.000	0.735
RISK–SZA	-0.160	0.151	1565	-1.061	-0.634	0.313	1.000	0.289
RISK–SZ	0.205	0.115	1565	1.78	-0.155	0.565	1.000	0.075
BDI–BDII	0.097	0.111	1565	0.87	-0.251	0.445	1.000	0.384
BDI–SZA	-0.014	0.117	1565	-0.122	-0.380	0.352	1.000	0.903
BDI–SZ	0.351	0.072	1565	4.89	0.126	0.575	3.12×10^{-5}	1.11×10^{-6}
BDII–SZA	-0.111	0.145	1565	-0.769	-0.563	0.341	1.000	0.442
BDII–SZ	0.254	0.112	1565	2.278	-0.095	0.603	0.640	0.023
SZA–SZ	0.365	0.117	1565	3.127	0.000	0.731	0.050	0.002

Note: The analyses accounted for age, sex, and the first five genetic ancestry principal components. Adjusted *p*, Bonferroni adjusted *p*-values per analysis; Beta, Beta Coefficient; CI, confidence interval; *df*, degrees of freedom; Est, Estimate; SE, Standard Error. Group abbreviations: BD I, bipolar I disorder; BD II, bipolar II disorder; CTRL, controls; RISK, at-risk state for psychosis; SCHIZ L, lower schizotypy; SCHIZ H, higher schizotypy; SZA, schizoaffective disorder; SZ, schizophrenia.

Table S3. Binary logistic regression for case–control contrasts across the polygenic risk scores (PRS) for schizophrenia and bipolar disorder.

Measure pair	Baseline R^2	Full R^2	$R^2\Delta$	p $R^2\Delta$	OR	95% CI
**Polygenic risk score for schizophrenia (SZ-PRS)**						
SZ–CTRL	0.136	0.194	0.057	6.88×10^{-9}***	1.624	1.374–1.930
SZA–CTRL	0.191	0.252	0.061	2.04×10^{-5}***	1.823	1.375–2.454
BD II–CTRL	0.129	0.130	0.002	0.500	1.093	0.845–1.419
BD I–CTRL	0.151	0.164	0.013	0.006**	1.268	1.070–1.508
RISK–CTRL	0.560	0.576	0.016	0.006**	1.571	1.139–2.203
SCHIZ H–CTRL	0.226	0.228	0.002	0.440	0.909	0.713–1.158
SCHIZ L–CTRL	0.267	0.268	<0.001	0.773	1.038	0.804–1.346
**Polygenic risk score for bipolar disorder (BD-PRS)**						
SZ–CTRL	0.136	0.148	0.011	0.011*	1.235	1.050–1.456
SZA–CTRL	0.191	0.263	0.072	3.41×10^{-6}***	1.959	1.464–2.665
BD II–CTRL	0.129	0.175	0.047	1.98×10^{-4}***	1.649	1.263–2.175
BD I–CTRL	0.151	0.228	0.077	2.53×10^{-11}***	1.797	1.505–2.162
RISK–CTRL	0.560	0.600	0.040	8.21×10^{-6}***	2.186	1.533–3.201
SCHIZ H–CTRL	0.226	0.228	0.003	0.340	1.137	0.874–1.485
SCHIZ L–CTRL	0.267	0.269	0.001	0.501	1.090	0.848–1.406

Note: The reported estimates of change (Δ) in R^2 represent the gain from adding polygenic risk score (Full R^2) to the model with covariates only (age, sex, five genetic ancestry principal components; Baseline R^2) in a block-wise binary regression. Significant effects from adding PRS to the model are marked with asterisks (*, $p < 0.05$; **, $p < 0.01$; ***, $p < 0.001$). CI, Confidence Interval. Group abbreviations: BD I, bipolar I disorder; BD II, bipolar II disorder; CTRL, controls; RISK, at-risk state for psychosis; SCHIZ H, higher schizotypy; SCHIZ L, lower schizotypy; SZA, schizoaffective disorder; SZ, schizophrenia.

Table S4. Results of the quantile regression analysis of polygenic risk scores (PRS) predicting symptoms.

Measure	Quantile	Coefficient	SE	*T*-statistic	*p*
**Polygenic risk score for schizophrenia (SZ-PRS)**					
PANSS Positive Symptoms	tau = 0.25	0.000	1.23×10^{29}	0.000	1.000
	tau = 0.50	3.351	0.690	4.859	1.35×10^{-6}***
	tau = 0.75	4.291	1.715	2.502	0.013*
PANSS Negative Symptoms	tau = 0.25	0.000	0.355	0.000	1.000
	tau = 0.50	3.260	1.234	2.643	0.008**
	tau = 0.75	3.376	2.327	1.451	0.147
PANSS General Psychopathology	tau = 0.25	3.225	0.946	3.410	6.70×10^{-4}***
	tau = 0.50	4.895	2.158	2.269	0.023*
	tau = 0.75	4.185	2.686	1.558	0.120
PANSS Total Score	tau = 0.25	6.128	1.586	3.864	1.18×10^{-4}***
	tau = 0.50	11.725	3.681	3.185	0.001**
	tau = 0.75	13.791	5.222	2.641	0.008**
**Polygenic risk score for bipolar disorder (BD-PRS)**					
PANSS Positive Symptoms	tau = 0.25	0.000	9.51×10^{28}	0.000	1.000
	tau = 0.50	-3.291	2.424	-1.357	0.175
	tau = 0.75	0.650	4.307	0.151	0.880
PANSS Negative Symptoms	tau = 0.25	0.000	0.717	0.000	1.000
	tau = 0.50	2.608	3.268	0.798	0.425
	tau = 0.75	-2.869	5.645	-0.508	0.611
PANSS General Psychopathology	tau = 0.25	5.776	3.565	1.620	0.105
	tau = 0.50	2.821	5.407	0.522	0.602
	tau = 0.75	-0.904	6.687	-0.135	0.893
PANSS Total Score	tau = 0.25	5.776	3.565	1.620	0.105
	tau = 0.50	2.821	5.407	0.522	0.602
	tau = 0.75	-0.904	6.687	-0.135	0.893

Note: The analyses accounted for age, sex and the first five genetic ancestry principal components. *, $p < 0.05$; **, $p < 0.01$; ***, $p < 0.001$. Abbreviations: PANSS, Positive and Negative Syndrome Scale; *p*, *p*-value; SE, Standard Error.

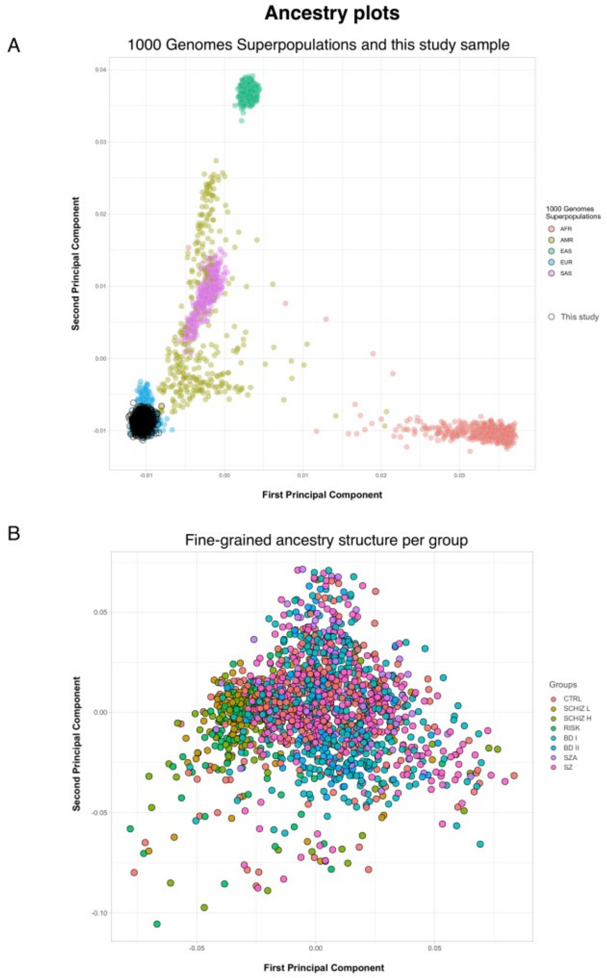

Figure S1. Plots of the first two ancestry principal components (A) overlaid onto the 1000 Genomes Project[1] global reference superpopulations and (B) according to study group. The plots confirm the homogeneous European origin of the study sample. Population codes: AFR, African; AMR, Admixed American; CEU, Utah Residents of Northern and Western European Ancestry; EAS, East Asian; EUR, European; FIN, Finnish in Finland; GBR, British in England and Scotland; IBS, Iberian population in Spain; SAS, South Asian; TSI, Tuscany, Italy. Group abbreviations: BD I, bipolar I disorder; BD II, bipolar II disorder; CTRL, control; RISK, at-risk state for psychosis; SCHIZ H, higher schizotypy; SCHIZ L, lower schizotypy; SZA, schizoaffective disorder; SZ, schizophrenia.

Supplementary References:

1. Schultze-Lutter F, Michel C, Schmidt SJ, Schimmelmann BG, Maric NP, Salokangas R, et al. EPA guidance on the early detection of clinical high risk states of psychoses. European Psychiatry. 2015;30(3):405-16.

2. Schultze-Lutter F, Addington J, Ruhrmann S, Klosterkötter J. Schizophrenia proneness instrument, adult version (SPI-A). Rome: Giovanni Fioriti. 2007.

3. McGlashan T, Miller T, Woods S, Rosen J, Hoffman R, Davidson L. Structured interview for prodromal syndromes. PRIME Research Clinic, Yale School of Medicine, New Haven, CT. 2001.

4. Raine A. The SPQ: a scale for the assessment of schizotypal personality based on DSM-III-R criteria. Schizophrenia bulletin. 1991;17(4):555-64.

5. Sheehan DV, Lecrubier Y, Sheehan KH, Amorim P, Janavs J, Weiller E, et al. The Mini-International Neuropsychiatric Interview (M.I.N.I.): the development and validation of a structured diagnostic psychiatric interview for DSM-IV and ICD-10. Journal of Clinical Psychiatry. 1998;59 Suppl 20:22-33.

6. Margraf J. Mini-DIPS: diagnostisches kurz-interview bei psychischen störungen: Springer-Verlag; 2013.

7. First MB. Structured clinical interview for the DSM (SCID). The encyclopedia of clinical psychology. 2014:1-6.

8. Kaufman J, Birmaher B, Brent D, Rao U, Flynn C, Moreci P, et al. Schedule for affective disorders and schizophrenia for school-age children-present and lifetime version (K-SADS-PL): initial reliability and validity data. Journal of the American Academy of Child & Adolescent Psychiatry. 1997;36(7):980-8.
